# Early versus late ultrasound and MRI fetal imaging for predicting outcomes in left‐sided congenital diaphragmatic hernia

**DOI:** 10.1002/pmf2.70119

**Published:** 2025-09-29

**Authors:** Lamia A. Alamri, Laura K. Kaizer, Jason Gien, S. Christopher Derderian, Mary D. Sammel, Mariana L. Meyers, Michael V. Zaretsky, Henry L. Galan

**Affiliations:** ^1^ Colorado Fetal Care Center Children's Hospital of Colorado Aurora Colorado USA; ^2^ Department of Obstetrics and Gynecology University of Colorado School of Medicine Aurora Colorado USA; ^3^ Department of Biostatistics and Informatics University of Colorado School of Medicine Aurora Colorado USA; ^4^ Department of Pediatrics University of Colorado School of Medicine Aurora Colorado USA; ^5^ Department of Pediatric Surgery University of Colorado School of Medicine Aurora Colorado USA; ^6^ Department of Pediatric Radiology University of Colorado School of Medicine Aurora Colorado USA

**Keywords:** CDH, congenital diaphragmatic hernia, ECMO, MRI, prenatal diagnosis, ultrasound

## Abstract

**Introduction:**

Prenatal ultrasound (US) and magnetic resonance imaging (MRI) prognostic measurements are routinely used to predict congenital diaphragmatic hernia (CDH) severity. Practice variability exists in the type and timing of measurements obtained. The primary objective of this study was to determine the best prenatal prognostic measures to predict the need for extracorporeal membrane oxygenation (ECMO). Similarly, the secondary objective was to determine which measures best predicted a variety of other neonatal outcomes.

**Methods:**

This is a retrospective study of left‐sided CDH affected pregnancies at a single fetal care center from 2013 to 2023 who had at least one US and MRI. The cohort was divided into early (24 week) and late (34 week) groups, based on gestational age at the time of evaluation. Lung‐to‐head ratio (LHR), observed‐to‐expected LHR (O/E LHR), O/E total lung volume, and percent predicted lung volume (PPLV) were obtained at each visit. Clinical data were obtained from the electronic medical record. Pregnancies with other major anomalies, genetic syndromes, and <32 weeks gestation at the time of delivery were excluded. To identify the prenatal measures most predictive of ECMO, logistic regression models were estimated using standardized measures as covariates, and the standardized odds ratios were compared across measures. Following the identification of the strongest predictive measure for each imaging modality, each measure from both timepoints was included in one model to identify the best time (early vs. late) for outcome prediction.

**Results:**

Of the 124 patients affected with CDH, 90 met inclusion criteria. 11 out of the 90 neonates did not survive to discharge (12.2%). Patients on ECMO were more likely to be female (59% *p* = 0.034) and have lower birthweight (2810 [413] vs. 3080 [483], *p* = 0.014). All prenatal measures between MRI and US were significantly associated with ECMO use; however, MRI PPLV and US O/E LHR at the late evaluation had the strongest association.

**Conclusion:**

In this cohort of left‐sided CDH, all prenatal prognostic measures by US and MRI were highly predictive of ECMO use. O/E LHR by US and PPLV by MRI were the best predictors of ECMO in newborns, and these were most predictive in late gestational age.

## 1 BACKGROUND

Congenital diaphragmatic hernia (CDH) results from failure of the diaphragm to close by 12 weeks of gestation with an incidence of 2.5/10,000 birth [1]. CDH results in herniation of intraabdominal contents into the thoracic cavity resulting in pulmonary hypoplasia and pulmonary hypertension (PH) leading to significant neonatal morbidity and mortality, including those related to extracorporeal membrane oxygenation (ECMO). Prenatal evaluation of CDH has notably evolved over the last few decades, allowing for improved patient counseling, fetal intervention in specific cases, and timely referral to specialized centers for optimal postnatal management.

Prenatal ultrasound (US) measurements of the lungs, including lung‐to‐head ratio (LHR), have been traditionally used for prognostication. While this measurement has been shown to independently predict neonatal outcomes, it comes with limitations, including a major critique that LHR values change exponentially with advancing gestational age (GA) [2]. This has incentivized the use of other prognostic measures that can be independent of or adjusted for GA such as the observed‐to‐expected LHR (O/E LHR) described by Jani et al. [3]. The O/E LHR has been shown to independently predict survival, with values <25% portending a poor neonatal prognosis and serving as the initial cutoff point for inclusion in the fetal tracheal balloon occlusion study [3, 4, 5]. Additionally, O/E LHR values remain relatively stable throughout gestation with good predictive value at early and later GA [6, 7]. Though, the use of O/E LHR is highly validated, it is still subject to variability related to differing acquisition techniques (i.e., manual trace vs. multiplication of longest perpendicular diameter) and to subjective interpretation of image quality [8].

Efforts to complement the use of US and to develop other accurate predictive measures have led to the use of magnetic resonance imaging (MRI), including percent predicted lung volume (PPLV), total lung volume (TLV) and observed‐to‐expected TLV (O/E TLV) in clinical practice [8, 9, 10, 11]. Third trimester MRI O/E TLV and PPLV have been shown to predict the need for ECMO [12, 13, 14]. However, there are still limitations to using MRI. For example, MRI PPLV values typically decrease over time, particularly when early GA values are greater than 15%, making a repeat MRI evaluation more helpful [12, 15]. Furthermore, the use of MRI can be prohibitively expensive, uncomfortable for patients, and/or time consuming for patients and physicians. Despite these limitations, fetal MRI has grown to be standard of care at many institutions across the country with improved accessibility, specifically in cases of CDH in order to acquire volumetric calculations that can have an impact on perinatal management. However, there is still some variability related to when fetal MRI is performed and if one or two are obtained at an early and late GA [16]. Other variability exists in the type of volumetric analyses performed by MRI at each institution. Some institutions only obtain MRI TLV, while others also obtain PPLV with or without O/E TLV.

These considerations have led to challenges faced by clinicians in identifying the best prognostic measurements and the most ideal timing to optimize the predictive value in CDH. A 2020 survey conducted across North American Fetal Therapy Network (NAFTNet) sites to evaluate antenatal prognostication of CDH found variability in US practices and even more disparity in MRI practice patterns as mentioned above [16].

Therefore, in this study, the primary aim was to ascertain which US and MRI prognostic measures were best in predicting the need for ECMO, with the ultimate goal of determining whether MRI at both timepoints is necessary. The secondary aim of this study was to determine which of the US and MRI prognostic measures are the best predictors of a variety of other neonatal outcomes. We hypothesize that both US and MRI will correlate highly with ECMO at earlier evaluations, but that MRI prognostic measures would perform the best as they assess both lung volumes.

## 2 METHODS

This retrospective study included singleton pregnancies complicated by CDH managed at an academic free‐standing fetal care center, from 2013 to 2023 who had at least one fetal MRI and one US performed during this period. Research ethics board approval was obtained by the University of Colorado multiple institutional review board (COMIRB #13‐1493). Prenatal and neonatal data were extracted from the electronic medical record. Those with right‐sided or bilateral CDH, additional major anomalies or confirmed genetic diagnoses, and who underwent fetoscopic endoluminal tracheal occlusion or delivered at an outside facility were excluded. Patients who were <32 weeks GA at birth were also excluded as they were not candidates for ECMO.

It is standard practice at our referral‐based fetal care center to image pregnancies complicated by CDH at two timepoints in pregnancy by both US and MRI typically done on the same day. The maximum separation in days when performed on different days was 1 week and this occurred in three cases. The initial US and MRI imaging evaluation is performed at approximately 24 weeks gestation, followed by repeat US and MRI at approximately 34 weeks. Each visit also has a multidisciplinary family consultation that includes a review of fetal growth and evaluation of CDH characteristics, including the prognostic measurements. CDH prognostic measures assessed included US LHR, O/E LHR and MRI PPLV, and O/E TLV. Due to a variety of clinical factors, time of initial referral and evaluation at our fetal care center, and remote patient residence, not all patients received imaging at both timepoints and there was some variation in GA at both timepoints.

### 2.1 Prenatal US measurements

**FIGURE 1:**
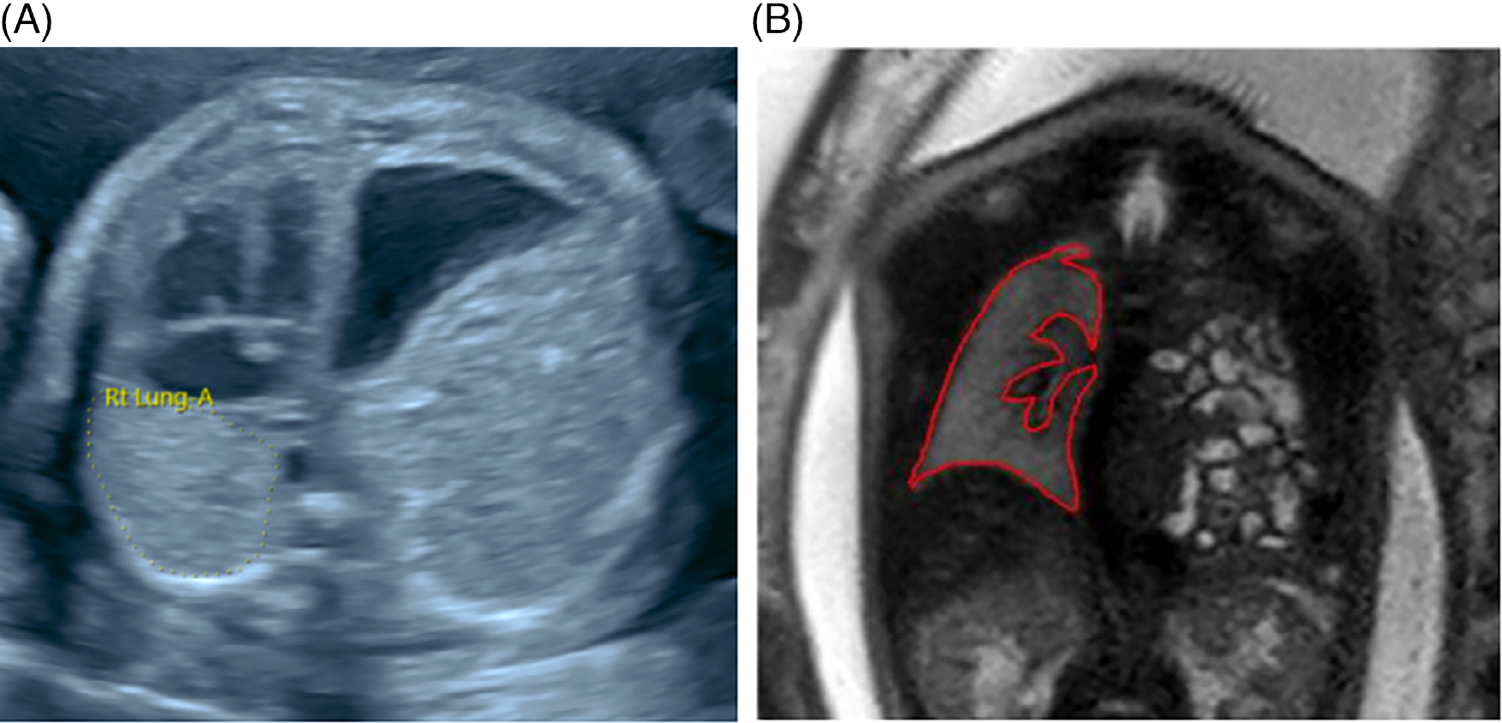
A 34‐week gestational age fetus with left‐sided congenital diaphragmatic hernia. (A) Axial ultrasound image of the fetal chest at the level of the four‐chamber heart view showing measurement of the contralateral lung via the trace method. (B) Coronal T2‐weighted single‐shot spin echo image in the same fetus showing how fetal lungs are contoured by magnetic resonance imaging (MRI) to obtain volumes.

The LHR measurements were performed by obtaining an axial view of the fetal chest at the level of the four‐chamber heart. The contralateral lung was measured via the trace method based on the NAFTNet measurement protocol [8]. Typically, three to six measurements are taken and averaged. O/E LHR was then obtained by dividing the observed LHR by the expected LHR using the Jani et al. method [3]. Images were obtained by trained sonographers with approximately 10–15 years of experience using GE Voluson E10 expert machines (GE Healthcare). The images were interpreted by one of three maternal fetal medicine specialist. Figure 1A shows a representative US image.

### 2.2 Prenatal MRI measurements

Noncontrast MR images were obtained on a 1.5 T scanner, either an Avanto (Siemens Healthcare) or an Ingenia (Philips Healthcare), using two 6‐channel surface coils or a 32‐channel surface coil, respectively. The images were acquired following our preexisting protocols for fetuses with suspected intrathoracic abnormalities. Protocols included no gap or overlap, and a slice thickness of 3–4 mm. No maternal sedation was used. Radiologists exported the best motion‐free coronal single‐shot fast spin echo T2‐weighted sequences of the fetal chest to a separate three‐dimensional workstation (Aquarius iNtuition edition 4.7; TeraRecon) to perform the volumetric analysis [13]. Figure 1B shows a representative MRI image.

### 2.3 Primary and secondary neonatal outcomes

The primary outcome was need for ECMO. The need for ECMO is determined by the level of required respiratory support and cardiac function in the delivery room. This includes failure to respond to gentle ventilation, preductal PaO2 < 40 mmHg, inability to maintain adequate blood pressure on vasopressors, persistent right to left atrial shunt, biventricular dysfunction, or those with signs of persistently impaired perfusion. Patients with severe US and MRI measurements are typically delivered via cesarean with ECMO on standby. ECMO would be initiated if they met criteria as outlined above. Survival to discharge was not included in our primary outcome, as mortality prior to discharge was relatively low in this cohort.

Secondary outcomes that were used in our analysis included, ventilatory days, (VD) which are total days to include brief periods of attempted extubation that later required reintubation; noninvasive ventilatory days (NIVD); days on inhaled nitric oxide (iNOD); duration of ECMO; and neonatal intensive care unit (NICU) length of stay (LOS). Medication administration was used as a surrogate for severity of PH as the decision to initiate and add PH therapy was based on echocardiographic findings, as well as clinically significant PH spells. PH severity was classified as follows: none—no need for medical management, mild—requiring only one medication (sildenafil), moderate—requiring two medications (sildenafil and bosentan) and severe—requiring three medications (sildenafil, bosentan, and prostacyclin analogue). Chronic lung disease (CLD) severity was classified as follows: none—no oxygen requirement at the time of NICU discharge, mild—requiring less than 0.5 lpm low flow nasal cannula (LFNC) at the time of NICU discharge, moderate—requiring more than 0.5 lpm LFNC at the time of NICU discharge, and severe—requiring tracheostomy prior to NICU discharge [17].

### 2.4 Statistical analysis

Mean (standard deviation), median (min, max), or *n* (percent) are presented, unless otherwise indicated. Differences in patient characteristics by ECMO were assessed using chi‐squared tests, Fisher's exact tests, Poisson regressions, and Wilcoxon rank sum tests, based on the distribution of each variable. Spearman correlations were calculated among all measures collected from each imaging modality (MRI and US). Variance inflation factors (VIF) were assessed at each step of modeling. Highly correlated measures (defined as having a VIF ≥ 5) were not included in the same model due to multicollinearity [18].

Due to the high degree of correlation between all measures, we took a stepwise approach to assess which were most strongly associated with ECMO. First, to be able to compare the odds ratios across measures with different units, all measures were standardized by calculating *z*‐scores. Individual logistic regression models, with ECMO as the outcome, were estimated. It was decided a priori to identify the strongest effects based on the smallest standardized odds ratios. As a sensitivity analysis, analyses were repeated among the subsample of patients who had both imaging modalities at both timepoints. Second, for the measures most strongly associated with ECMO from each imaging modality, one model was run with both timepoints, to assess whether both timepoints were significantly associated with ECMO when adjusting for the other. Finally, one model was selected that included the best measures from each imaging modality. The predictive accuracy was summarized using the area under the curve‐receiver operating characteristic curve (AUC‐ROC). This was calculated for each model separately. Due to the small sample size, AUC comparison tests were not performed. In order to identify a threshold for the strongest predictor of ECMO, the Youden's *J* index was calculated to identify the optimal cutoff value for the best predictor while maximizing sensitivity. Analyses were completed using SAS version 9.4 and R [19].

## 3 RESULTS

### 3.1 Study subject characteristics

**FIGURE 2:**
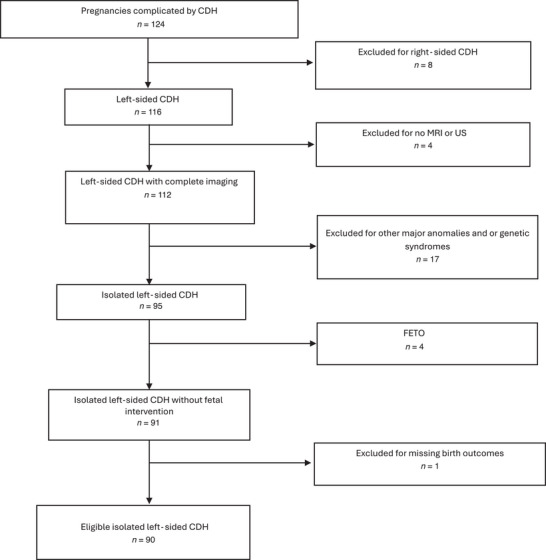
Flow diagram of patient selection. CDH, congenital diaphragmatic hernia; FETO, fetal endotracheal occlusion; MRI, magnetic resonance imaging; US, ultrasound.

**FIGURE 3:**
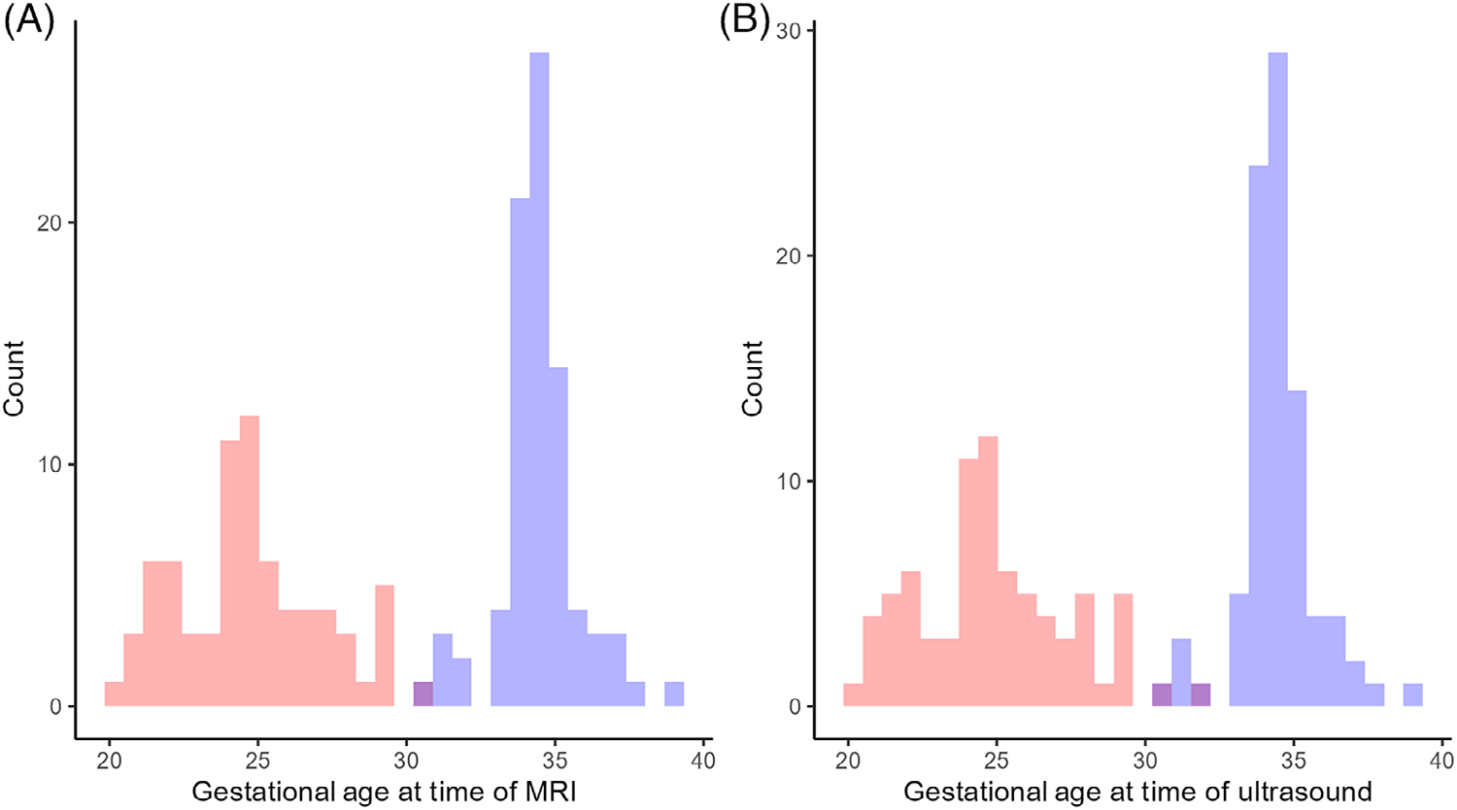
Gestational age distribution at time of early and late imaging evaluation. (A) Red: early magnetic resonance imaging (MRI) (*n* = 73, median 24 weeks), blue: late MRI (*n* = 83, median 34 weeks). (B) Red: early ultrasound (US) (*n* = 76, median 24 weeks), blue: late US (*n* = 84, median 34 weeks).

Of the 124 patients affected with CDH, 90 met inclusion criteria shown in Figure 2. Note that 11 out of the 90 neonates did not survive to discharge (12.2%). Table S1 provides a summary of LHR1 and O/E LHR1 measurements stratified by survival. Maternal and neonatal cohort characteristics are reported in Table 1. Patients on ECMO were more likely to be female (*p* = 0.034), have lower birthweight (*p* = 0.014), and lower rapid clinical assessment of newborn's physical status at birth (APGAR) scores (*p* = 0.003, *p* < 0.001). There were no statistically significant differences in ECMO by infant race, GA at delivery, maternal race, maternal age at delivery, or maternal body mass index. Figure 3 shows the distribution of GA at the early and late imaging evaluations.

**TABLE 1:** Characteristics of study patient population relative to extracorporeal membrane oxygenation (ECMO) use.

	No ECMO (*n* = 58)	ECMO (*n* = 32)	Overall (*n* = 90)	*p* value
Infant sex				0.034
Female	21 (36.2%)	19 (59.4%)	40 (44.4%)	
Male	37 (63.8%)	13 (40.6%)	50 (55.6%)	
Infant race				0.637
Hispanic/Latino	18 (31.0%)	8 (25.0%)	26 (28.9%)	
Other	3 (5.2%)	3 (9.4%)	6 (6.7%)	
White	37 (63.8%)	21 (65.6%)	58 (64.4%)	
Birth weight				0.014
Mean (SD)	3080 (483)	2810 (413)	2980 (475)	
Median [min, max]	3090 [2000, 4030]	2920 [2000, 3500]	3000 [2000, 4030]	
Maternal age at delivery				0.863
Mean (SD)	30.1 (5.66)	29.4 (6.21)	29.8 (5.83)	
Median [min, max]	30.0 [20.0, 42.0]	30.0 [17.0, 41.0]	30.0 [17.0, 42.0]	
Gestational age				0.162
Mean (SD)	38.0 (1.23)	37.6 (1.35)	37.8 (1.28)	
Median [min, max]	38.1 [33.6, 39.7]	38.0 [34.0, 39.3]	38.1 [33.6, 39.7]	
Maternal race				0.278
Hispanic/Latino	18 (31.0%)	8 (25.0%)	26 (28.9%)	
Other	2 (3.4%)	4 (12.5%)	6 (6.7%)	
White	34 (58.6%)	20 (62.5%)	54 (60.0%)	
Missing	4 (6.9%)	0 (0%)	4 (4.4%)	
Maternal BMI				0.810
Mean (SD)	30.1 (6.45)	30.5 (6.81)	30.3 (6.54)	
Median [min, max]	29.6 [20.0, 48.0]	31.0 [20.0, 49.0]	30.0 [20.0, 49.0]	
Missing	1 (1.7%)	1 (1.3%)	2 (2.2%)	
1‐min APGAR				0.003
Mean (SD)	5.43 (1.96)	4.09 (1.77)	4.96 (1.99)	
Median [min, max]	5.50 [1.00, 8.00]	4.00 [1.00, 7.00]	5.00 [1.00, 8.00]	
5‐min APGAR				<0.001
Mean (SD)	7.45 (1.43)	6.47 (1.29)	7.10 (1.45)	
Median [min, max]	8.00 [2.00, 9.00]	7.00 [3.00, 9.00]	7.00 [2.00, 9.00]	

*Note*: Differences between those with and without ECMO were assessed using chi‐squared tests, Fisher's exact tests, Poisson regressions, and Wilcoxon rank sum tests.

Abbreviation: APGAR, rapid clinical assessment of newborn status at birth.

**FIGURE 4:**
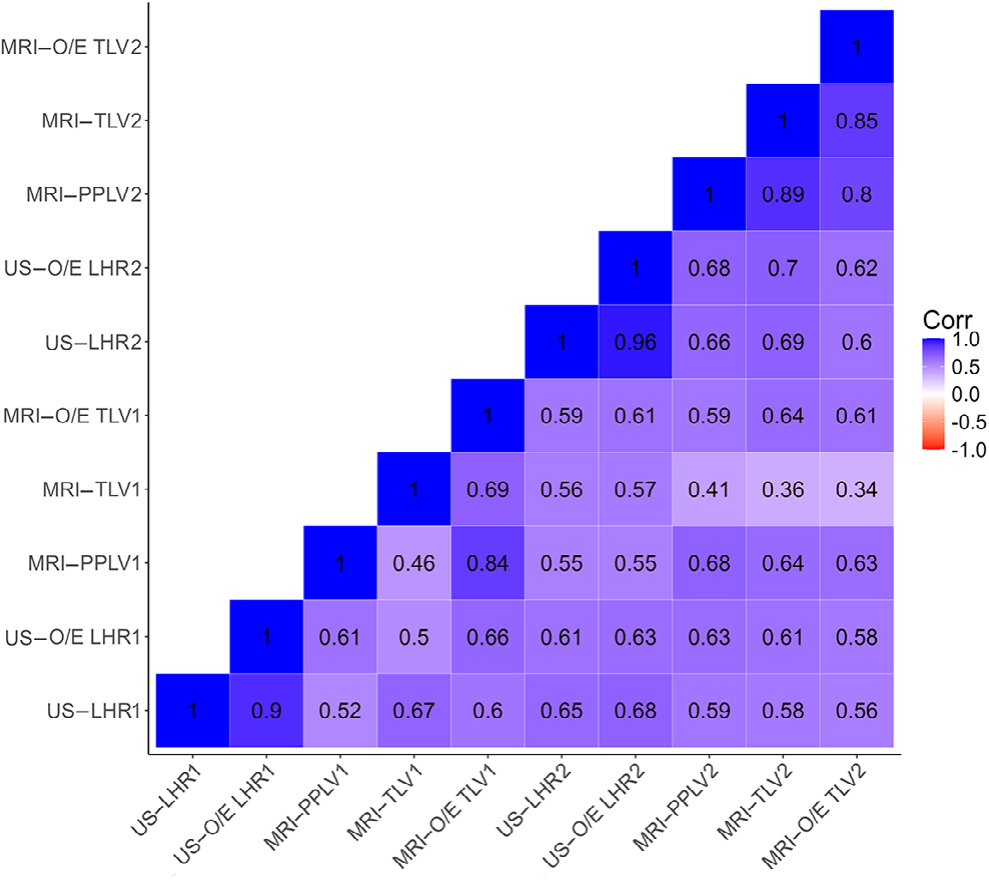
Significant Spearman correlations between pairs of prognostic measurement. LHR, lung‐to‐head ratio; MRI, magnetic resonance imaging; O/E LHR, observed‐to‐expected lung‐to‐head ratio; O/E TLV, observed‐to‐expected total lung volume; PPLV, percent predicted lung volume; TLV, total lung volume; US, ultrasound.

Table 2 provides a descriptive summary of primary and secondary outcomes for the cohort. Average and median values for each prognostic measurement in relation to ECMO use are reported in Table S2. Significant Spearman correlations between pairs of prognostic measurements are shown in Figure 4. All prognostic measurements were significantly correlated with each other. However, early MRI TLV and late MRI TLV and MRI O/E TLV had the weakest correlations.

**TABLE 2:** Descriptive summary of primary and secondary outcomes in the study cohort.

Clinical outcome	Overall (*n* = 90)
ECMO	
No	58 (64.4%)
Yes	32 (35.6%)
Survival to discharge	
No	11 (12.2%)
Yes	79 (87.8%)
NICU length of stay	
Mean (SD)	71.0 (70.4)
Median [min, max]	48.5 [1.00, 450]
Ventilatory days	
Mean (SD)	32.5 (58.0)
Median [min, max]	15.0 [0, 450]
Inhaled nitric oxide duration	
Mean (SD)	37.1 (51.4)
Median [min, max]	21.5 [0, 293]
Chronic lung disease	
None	18 (20.0%)
Mild	47 (52.2%)
Moderate	7 (7.8%)
Severe	7 (7.8%)
Missing	11 (12.2%)
Pulmonary hypertension	
None	43 (47.8%)
Mild	27 (30.0%)
Moderate	7 (7.8%)
Severe	2 (2.2%)
Missing	11 (12.2%)
Noninvasive respiratory support	
Mean (SD)	21.9 (28.2)
Median [min, max]	11.0 [0, 158]
Missing	11 (12.2%)
ECMO cannulation duration	
Mean (SD)	17.6 (14.7)
Median [min, max]	14.5 [1.00, 65.0]
Missing	58 (64.4%)

Abbreviations: ECMO, extracorporeal membrane oxygenation; NICU, neonatal intensive care unit.

### 3.2 Primary outcome

The associations between prenatal imaging measurements and the odds of ECMO use are described in Table 3. Importantly, all included prognostic measures were significantly and strongly associated with ECMO use. However, measures taken later in gestation outperformed their early counterparts. PPLV at the late timepoint had the strongest association with ECMO. On average, for every 1 unit increase in PPLV at the late GA evaluation, the odds of ECMO decreased by 22% (95%CI: 12%–30%). The AUC for this model was 0.86 (95% CI: 0.77, 0.94). MRI measures at the early GA evaluation (i.e., O/E TLV, PPLV, and TLV) showed the weakest association with ECMO use. These results were consistent among the subsample of patients who had both scans at both timepoints.

**TABLE 3:** Logistic regression for standardized measures with extracorporeal membrane oxygenation (ECMO) as dependent variable.

Prognostic measurement	*n*	^a^Odds ratio standardized scale	^b^Odds ratio original scale	Wald chi‐square	*p* value	Area under the curve
PPLV2	83	0.06 (0.01, 0.22)	0.78 (0.70, 0.88)	17.5452	<0.0001	0.86 (0.77, 0.94)
TLV2	83	0.10 (0.03, 0.29)	0.86 (0.80, 0.92)	16.6234	<0.0001	0.85 (0.77, 0.94)
O/E LHR2	84	0.11 (0.04, 0.32)	0.90 (0.86, 0.95)	16.736	<0.0001	0.87 (0.79, 0.95)
LHR2	84	0.12 (0.04, 0.33)	0.44 (0.30, 0.65)	16.9643	<0.0001	0.86 (0.77, 0.95)
LHR1	76	0.16 (0.06, 0.40)	0.30 (0.17, 0.55)	15.6349	<0.0001	0.83 (0.74, 0.92)
O/E LHR1	76	0.22 (0.11, 0.46)	0.90 (0.85, 0.95)	16.4055	<0.0001	0.81 (0.72, 0.91)
O/E TLV2	83	0.23 (0.10, 0.52)	0.92 (0.88, 0.97)	12.4983	0.0004	0.80 (0.70, 0.90)
O/E TLV1	73	0.27 (0.13, 0.56)	0.90 (0.85, 0.96)	12.3276	0.0004	0.77 (0.65, 0.88)
PPLV1	73	0.27 (0.13, 0.57)	0.86 (0.79, 0.94)	12.0829	0.0005	0.80 (0.69, 0.91)
TLV1	73	0.32 (0.15, 0.67)	0.82 (0.72, 0.93)	9.2682	0.0023	0.75 (0.63, 0.86)

*Note*: LHR is modeled per every 0.25 unit increase. Odds ratios can be interpreted as follows: For every 0.25 unit increase in LHR, the odds of ECMO decrease by (1 − OR)%. “1” denotes early evaluation, and “2” denotes late evaluation.

Abbreviations: LHR, lung‐to‐head ratio; O/E LHR, observed‐to‐expected lung‐to‐head ratio; O/E TLV, observed‐to‐expected total lung volume; PPLV, percent predicted lung volume; TLV, total lung volume.

^a^Logistic regression models using standardized (*z*‐score) measures. The odds ratios may be compared with each other across models.

^b^Logistic regression models using original measures. The odds ratios may *not* be compared with each other across models. The units are different across models.

Because PPLV at the late GA timepoint was the measure most strongly associated with ECMO, Youden's *J* index was calculated to identify a PPLV2 cutoff that would best predict ECMO use in this cohort. This method identified a threshold for PPLV2 of 17.85% for predicting ECMO. This threshold had a 90.3% sensitivity, a 66.0% specificity, with an accuracy of 75.0%.  When comparing the 17.85% threshold to the established clinical threshold of 15%, the 17.85% threshold identified additional six true ECMO cases, while also identifying four additional false positives.

Table 4 depicts the measurements most strongly associated with ECMO use from each imaging modality individually. In Model 1, when including both PPLV1 and PPLV2, PPLV2 remained significantly associated with ECMO (*p* = 0.026), while PPLV1 was no longer significant. Similarly, Model 2 included the measures from US, O/E LHR1, and O/E LHR2, only O/E LHR2 remained significantly associated with ECMO (*p* = 0.008).

**TABLE 4:** Combined logistic regression model for extracorporeal membrane oxygenation (ECMO) with most strongly associated ultrasound (US) and magnetic resonance imaging (MRI) measurements at both timepoints.

Model	*n*	Prognostic measurement	Odds ratio	Wald chi‐square	*p* value	AUC
Model 1	66	PPLV1 PPLV2	0.46 (0.19, 1.13) 0.17 (0.04, 0.81)	2.8359 4.944	0.092 0.026	0.82 (0.72, 0.93)
Model 2	70	O/E LHR1 O/E LHR2	0.46 (0.18, 1.16) 0.20 (0.06, 0.66)	2.707 6.9802	0.100 0.008	0.86 (0.78, 0.95)
Model 3	80	O/E LHR2 PPLV2	0.21 (0.07, 0.63) 0.20 (0.04, 0.87)	7.7676 4.5821	0.005 0.032	0.89 (0.81, 0.97)

*Note*: “1” denotes early evaluation, and “2” denotes late evaluation.

Abbreviations: AUC, area under the curve; O/E LHR, observed‐to‐expected lung‐to‐head ratio; PPLV, percent predicted lung volume.

Finally, Model 3 shows the relationship between the best MRI and US measurements at Timepoint 2 (PPLV2 and O/E LHR2). In this combined model, both O/E LHR2 and PPLV2 were significantly associated with ECMO (*p* = 0.005 and *p* = 0.032, respectively).

### 3.3 Secondary outcomes

**FIGURE 5:**
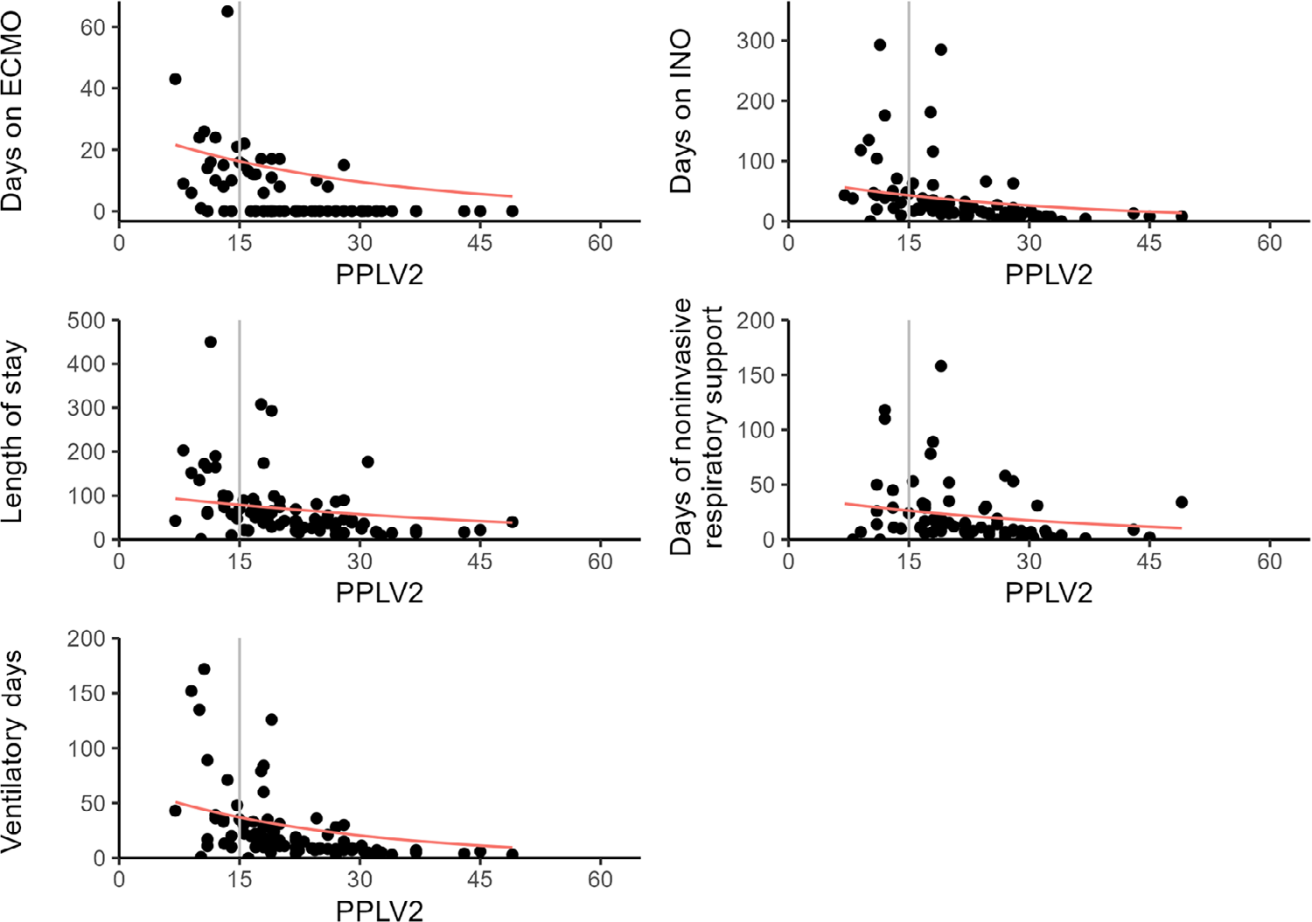
Associations between percent predicted lung volume (PPLV2) and secondary outcomes, with predicted lines from negative binomial regression models shown in red. ECMO, extracorporeal membrane oxygenation; INO, inhaled nitric oxide.

**FIGURE 6:**
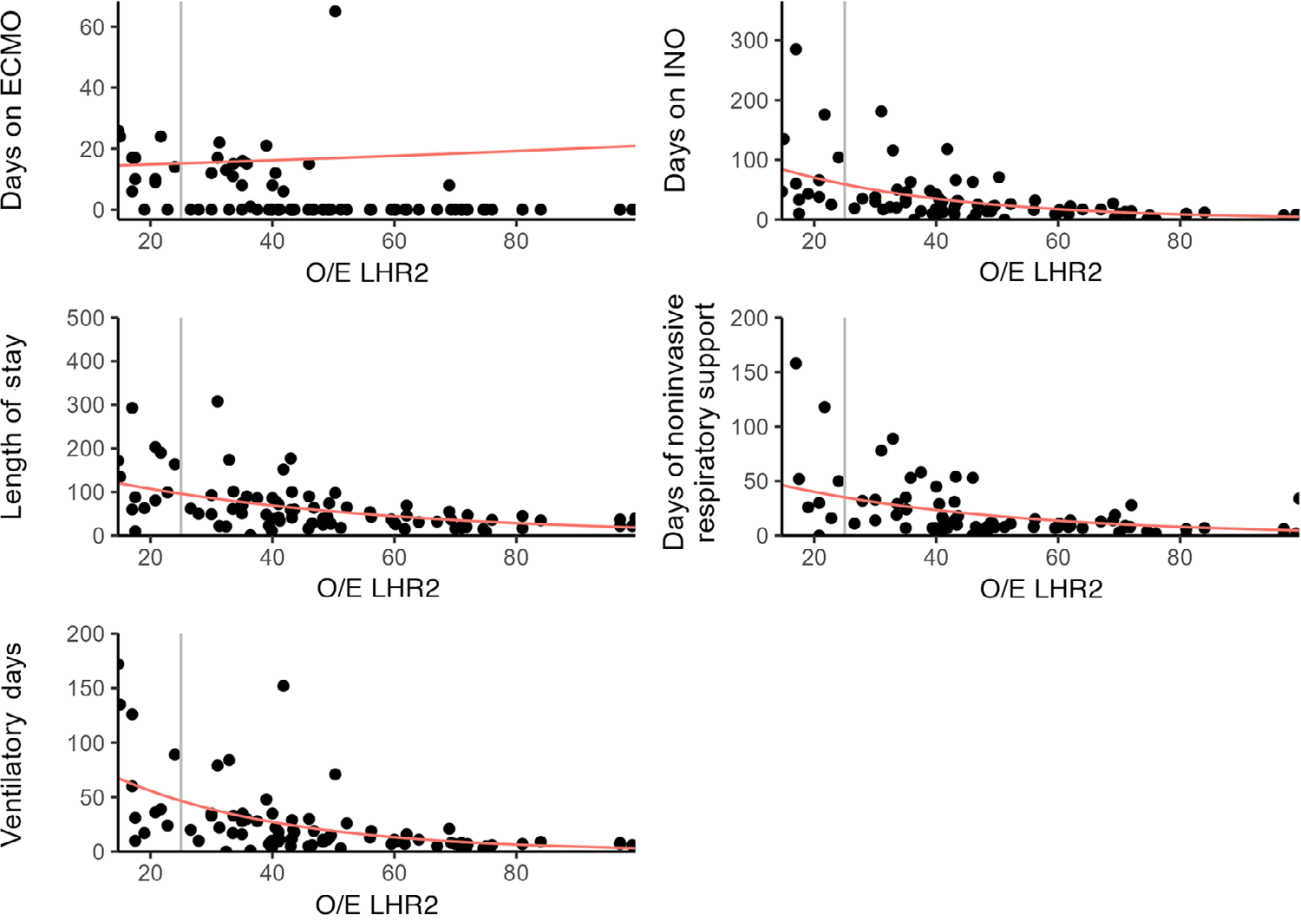
Associations between observed‐to‐expected lung‐to‐head ratio (O/E LHR2) and secondary outcomes, with predicted lines from negative binomial regression models shown in red. ECMO, extracorporeal membrane oxygenation INO, inhaled nitric oxide.

All the prognostic measures were significantly associated with LOS, VD, iNOD, and NIVD (Tables S3 and S4). All except for O/E TLV1 and PPLV1 were associated with CLD. All except for TLV1 were associated with PH. Only PPLV1 was significantly associated with ECMO days (*p* = 0.035). However, the analyses of CLD, PH, and ECMO days were limited due to either small sample or small group sizes. Because PPLV2 and O/E LHR2 were the strongest measures associated with ECMO, Figures 5 and 6 were constructed to demonstrate the overall inverse relationship between these measures and secondary outcomes LOS, VD, iNOD, and NIVD.

## 4 DISCUSSION

In this group of fetuses with isolated left CDH, all US and MRI prognostic measurements were found to be significantly associated with ECMO use. The high overall survival rate of 87.8% for this cohort limits the use of US and MRI prognostic assessment for survival and is the reason for using ECMO as the primary endpoint. Furthermore, PPLV and O/E LHR from the late GA evaluation had the strongest associations with ECMO. While we typically use a traditional PPLV threshold of <15% to mobilize ECMO standby at the time of delivery, our results identify a slightly higher cutoff of 17.85% that was able to predict additional six cases that ultimately required ECMO [9]. The results did show a female predominance in the ECMO group. Historically, female sex has been associated with improved outcomes in the NICU; however, this advantage tends to diminish with advancing GAs. With this cohort, the average GA at delivery was 38.0 (SD: 1.23) and 38.0 (SD: 1.23) in the non‐ECMO and ECMO groups, respectively. Neonates that required ECMO were more likely to have lower birth weights. On average, this reflected a 270 g difference between the two groups, which is statistically significant but unlikely to be clinically significant. This difference was unrelated to GA at delivery. There may be an association between small for gestational age neonates and worse CDH outcomes, although this was not directly assessed in this study.

Practice variability includes technical details such as differing LHR acquisition methods, variability in GA at time of US, and the use of MRI as an adjunct tool. As such, variations in imaging practices can limit the conduct and interpretation of studies. This study provides the advantage of examining a standardized approach with a more contemporary and relatively low mortality cohort in the prenatal diagnosis of CDH over a 10‐year period. When considering each prognostic measurement separately, it is important to note that all measurements were highly predictive of ECMO use, regardless of modality (MRI vs. US), or timing in pregnancy (Table 3). However, late time GA imaging, specifically PPLV2 and O/E LHR2 had the highest association with need for ECMO.

Optimal timing for MRI‐derived lung volumes has been inconsistently described in the literature depending on the specific prognostic measurements examined. In a retrospective cohort of 256 fetuses with CDH, Style et al. showed that third trimester MRI‐derived lung volumes were independent predictors of need for ECMO [15]. In a retrospective cohort of 354 patients with isolated CDH, Dutemeyer et al. specifically examined O/E TLV at different GA timepoints and found that prediction accuracy for need for ECMO was comparable starting at the mid second trimester [20]. Similar to our findings, a retrospective cohort study of isolated CDH by Madenci et al. showed that while LHR, O/E LHR, and PPLV were highly associated with ECMO, PPLV was a more discriminative measure [21]. This was also the conclusion of a study by Niemiec et al., noting that PPLV at 34 weeks GA is the best independent predictor of in‐hospital mortality and ECMO use [13]. It has also been proposed that assessment of fetal lung volumes later in gestation is a more accurate reflection of lung growth that should have already occurred [22]. For these reasons, at our institution, we have historically relied more heavily on the PPLV for measuring lung volumes than other MRI parameters. Interestingly, early MRI evaluation prognostic measurements from this study showed the weakest association with the need for ECMO. While this questions the utility of MRI in this early timepoint, it may provide information that influences reproductive choices.

Recognizing the most reliable prognostic measurements has important implications for family counseling and providing the appropriate antepartum and postnatal management. These data indicate that early imaging provides accurate predictions for disease severity. Providing this information is important, particularly for families who choose not to continue a pregnancy. Since all prognostic measures are comparable in their ability to predict morbidity, our findings suggest that reserving MRI evaluation for later GA may be reasonable. This would allow for detailed evaluation of lung volumes and decrease the health care costs associated with multiple MRI scans. This is further supported by the fact that early MRI measurements were the weakest predictive of ECMO. It is also reassuring to know that patients presenting at later GA will be afforded this benefit.

It is important to note that data from this study may be applied broadly to other specialized fetal centers. While the application of our findings to other centers may be limited by differences in neonatal practice and care of the CDH newborn, they may be more specifically applicable to prenatal imaging and evaluation. While the sample size of this study is large for a single center, additional multicenter studies would be beneficial to reexamine the difference in predictive value for ECMO use between both US and MRI at different timepoints. The retrospective nature of the study comes with inherent limitations including missing or incomplete data including inconsistent reporting on additional prenatal predictive markers such as stomach location and percent liver herniation. The majority of MRI and US imaging studies were nearly universally conducted on the same day with just a few that differed by a few days. While the median GAs at the early and late timepoint imaging were clustered around 24 and 34 weeks per protocol, there was a wider range in GA depending on timing of referral. Additionally, the number of MRI scans was higher in the late gestational time group compared to the early time group (83 vs. 73), which may introduce some bias in interpretation of the data.

In conclusion, our study demonstrates significant associations between the most utilized US and MRI prenatal prognostic measurements with ECMO use and a variety of other secondary outcomes. While all prognostic factors were highly predictive of ECMO use, PPLV by MRI and O/E LHR by US had the best correlation. Prognostic measures derived from imaging studies conducted in the late third trimester showed the strongest association. To our knowledge, this is one of the few studies that has compared both US and MRI prognostic measurements at different timepoints in gestation in left‐sided CDH. Multicenter studies with larger sample sizes from different practice settings can be used to validate the findings of this study and to confirm the higher cutoff of 17.85% for PPLV2 to predict severe left‐sided CDH.

## AUTHOR CONTRIBUTIONS

*Conceptualization*: Lamia A. Alamri, S. Christopher Derderian, Henry L. Galan, and Michael V. Zaretsky. *Methodology*: Laura K. Kaizer, Mary D. Sammel, Henry L. Galan, and Lamia A. Alamri. *Investigation*: Jason Gien, Henry L. Galan, and Lamia A. Alamri. *Data curation*: Lamia A. Alamri. *Writing—original draft*: Lamia A. Alamri. *Writing—review and editing*: S. Christopher Derderian, Jason Gien, Mariana L. Meyers, Michael V. Zaretsky, and Henry L. Galan, Laura K. Kaizer. *Supervision*: Henry L. Galan, Michael V. Zaretsky, Mariana L. Meyers, and Jason Gien.

## CONFLICT OF INTEREST STATEMENT

The authors declare no conflicts of interest.

## FUNDING INFORMATION

The authors received no specific funding for this work.

## ETHICS STATEMENT

A research ethics board approval was obtained by the University of Colorado COMIRB protocol #13‐1493.

## Supporting information



Supporting Information

## Data Availability

The data that support the findings of this study are not publicly available due to concerns for subject confidentiality but are available from the corresponding author upon reasonable request.
