# Supplementary material for: Early versus late ultrasound and MRI fetal imaging for predicting outcomes in left‐sided congenital diaphragmatic hernia
Source: Pregnancy (Hoboken). 2025 Sep 29;1(6):e70119. doi: 10.1002/pmf2.70119 (PMC13344685; doi:10.1002/pmf2.70119)
Supplement: Supplementary file 1 [file PMF2-1-e70119-s001.docx]

**Supplemental**

**Table 1** Summary of CDH severity by survival

|  | Died (N=11/90) | Survived (N=79) | Overall (N=90) |
| --- | --- | --- | --- |
| **LHR1 categories** |  |  |  |
| LHR1 =<1 | 10 (23.8%) | 32 (76.2%) | 42 |
| LHR1>1 | 1 (2.9%) | 33 (97.1%) | 34 |
| Missing | 0 | 14 | 14 |
| **OE_LHR1 categories** |  |  |  |
| O/E LHR 1 <25 | 4 (57.1%) | 3 (42.9%) | 7 |
| O/E LHR 1>=25 | 7 (10.1%) | 62 (89.9%) | 69 |
| Missing | 0 | 14 | 14 |

Percents displayed are row percents.

**Table 2**  Average and median MRI and US measurements at the two different time points in relation to ECMO use

|  | No ECMO  (n=58) | ECMO  (n=32) | Overall  (n=90) |
| --- | --- | --- | --- |
| **PPLV1** |  |  |  |
| Mean (SD) | 27.2 (8.01) | 19.3 (7.15) | 23.9 (8.55) |
| Median [Min, Max] | 24.0 [16.0, 53.0] | 18.0 [8.00, 36.0] | 22.0 [8.00, 53.0] |
| Missing | 15 (25.9%) | 2 (6.3%) | 17 (18.9%) |
| **TLV1** |  |  |  |
| Mean (SD) | 13.9 (6.25) | 9.25 (3.92) | 12.0 (5.85) |
| Median [Min, Max] | 11.8 [4.80, 34.0] | 8.28 [3.90, 22.0] | 10.3 [3.90, 34.0] |
| Missing | 15 (25.9%) | 2 (6.3%) | 17 (18.9%) |
| **O/E_TLV1** |  |  |  |
| Mean (SD) | 43.5 (12.1) | 31.7 (10.6) | 38.7 (12.9) |
| Median [Min, Max] | 42.8 [22.3, 81.4] | 31.5 [14.1, 56.6] | 37.8 [14.1, 81.4] |
| Missing | 15 (25.9%) | 2 (6.3%) | 17 (18.9%) |
| **PPLV2** |  |  |  |
| Mean (SD) | 26.1 (12.6) | 15.3 (5.13) | 22.2 (11.7) |
| Median [Min, Max] | 24.0 [11.0, 95.0] | 14.9 [7.00, 28.0] | 19.3 [7.00, 95.0] |
| Missing | 5 (8.6%) | 2 (6.3%) | 7 (7.8%) |
| **TLV2** |  |  |  |
| Mean (SD) | 39.7 (15.6) | 23.0 (7.98) | 33.7 (15.6) |
| Median [Min, Max] | 34.0 [13.0, 87.2] | 23.2 [11.7, 40.1] | 31.6 [11.7, 87.2] |
| Missing | 5 (8.6%) | 2 (6.3%) | 7 (7.8%) |
| **O/E_TLV2** |  |  |  |
| Mean (SD) | 43.6 (19.7) | 26.9 (9.39) | 37.5 (18.5) |
| Median [Min, Max] | 39.9 [0.600, 99.0] | 25.9 [11.7, 48.2] | 34.1 [0.600, 99.0] |
| Missing | 5 (8.6%) | 2 (6.3%) | 7 (7.8%) |
| **LHR1** |  |  |  |
| Mean (SD) | 1.25 (0.362) | 0.825 (0.253) | 1.08 (0.383) |
| Median [Min, Max] | 1.15 [0.520, 2.10] | 0.800 [0.400, 1.30] | 1.00 [0.400, 2.10] |
| Missing | 13 (22.4%) | 1 (3.1%) | 14 (15.6%) |
| **O/E_LHR1** |  |  |  |
| Mean (SD) | 50.3 (12.4) | 34.7 (11.7) | 43.9 (14.3) |
| Median [Min, Max] | 51.0 [29.3, 76.0] | 36.5 [14.5, 55.0] | 43.7 [14.5, 76.0] |
| Missing | 13 (22.4%) | 1 (3.1%) | 14 (15.6%) |
| **LHR2** |  |  |  |
| Mean (SD) | 1.73 (0.621) | 1.02 (0.384) | 1.50 (0.648) |
| Median [Min, Max] | 1.60 [0.700, 3.40] | 1.00 [0.470, 2.10] | 1.40 [0.470, 3.40] |
| Missing | 1 (1.7%) | 5 (15.6%) | 6 (6.7%) |
| **O/E_LHR2** |  |  |  |
| Mean (SD) | 55.4 (19.5) | 31.4 (12.6) | 47.7 (20.8) |
| Median [Min, Max] | 49.6 [19.0, 99.5] | 32.4 [14.7, 69.0] | 43.2 [14.7, 99.5] |
| Missing | 1 (1.7%) | 5 (15.6%) | 6 (6.7%) |

**Table 3** Negative binomial regression models for secondary count outcomes

| Outcome | Measure | n | Rate Ratio (95% CI) | p-value |
| --- | --- | --- | --- | --- |
| **ECMO Cannulation (days)** | PPLV2 | 30 | 0.66 (0.40, 1.09) | 0.1021 |
|  | PPLV1 | 30 | 0.75 (0.57, 0.98) | 0.0346 |
|  | O_E_TLV_1 | 30 | 0.77 (0.59, 1.02) | 0.0674 |
|  | TLV1 | 30 | 0.79 (0.54, 1.15) | 0.2146 |
|  | O_E_TLV_2 | 30 | 0.79 (0.51, 1.23) | 0.3008 |
|  | LHR1 | 31 | 0.80 (0.56, 1.13) | 0.2008 |
|  | TLV2 | 30 | 0.81 (0.52, 1.26) | 0.3462 |
|  | O_E_LHR_1 | 31 | 0.84 (0.64, 1.12) | 0.2343 |
|  | O_E_LHR_2 | 27 | 1.09 (0.75, 1.59) | 0.6406 |
|  | LHR2 | 27 | 1.14 (0.80, 1.64) | 0.4725 |
| **Inhaled Nitric Oxide (days)** | TLV2 | 83 | 0.48 (0.40, 0.58) | <.0001 |
|  | O_E_LHR_2 | 84 | 0.48 (0.41, 0.58) | <.0001 |
|  | O_E_LHR_1 | 76 | 0.50 (0.41, 0.61) | <.0001 |
|  | LHR2 | 84 | 0.51 (0.43, 0.61) | <.0001 |
|  | O_E_TLV_2 | 83 | 0.51 (0.42, 0.63) | <.0001 |
|  | LHR1 | 76 | 0.52 (0.42, 0.63) | <.0001 |
|  | O_E_TLV_1 | 73 | 0.52 (0.42, 0.65) | <.0001 |
|  | TLV1 | 73 | 0.56 (0.44, 0.71) | <.0001 |
|  | PPLV1 | 73 | 0.56 (0.45, 0.71) | <.0001 |
|  | PPLV2 | 83 | 0.67 (0.58, 0.79) | <.0001 |
| **Length of Stay** | LHR1 | 76 | 0.62 (0.53, 0.72) | <.0001 |
|  | TLV2 | 83 | 0.62 (0.54, 0.72) | <.0001 |
|  | O_E_LHR_2 | 84 | 0.63 (0.56, 0.72) | <.0001 |
|  | LHR2 | 84 | 0.63 (0.56, 0.72) | <.0001 |
|  | O_E_LHR_1 | 76 | 0.67 (0.57, 0.79) | <.0001 |
|  | O_E_TLV_2 | 83 | 0.67 (0.57, 0.78) | <.0001 |
|  | O_E_TLV_1 | 73 | 0.71 (0.60, 0.84) | <.0001 |
|  | PPLV1 | 73 | 0.72 (0.61, 0.86) | 0.0002 |
|  | TLV1 | 73 | 0.72 (0.61, 0.87) | 0.0004 |
|  | PPLV2 | 83 | 0.78 (0.69, 0.88) | <.0001 |
| **Non-invasive respiratory support(days)** | LHR1 | 65 | 0.50 (0.40, 0.62) | <.0001 |
|  | O_E_LHR_1 | 65 | 0.53 (0.41, 0.67) | <.0001 |
|  | O_E_TLV_2 | 73 | 0.55 (0.43, 0.70) | <.0001 |
|  | O_E_LHR_2 | 75 | 0.57 (0.48, 0.68) | <.0001 |
|  | TLV2 | 73 | 0.57 (0.46, 0.72) | <.0001 |
|  | O_E_TLV_1 | 62 | 0.58 (0.44, 0.76) | <.0001 |
|  | TLV1 | 62 | 0.59 (0.47, 0.74) | <.0001 |
|  | LHR2 | 75 | 0.60 (0.50, 0.72) | <.0001 |
|  | PPLV1 | 62 | 0.64 (0.49, 0.84) | 0.0011 |
|  | PPLV2 | 73 | 0.73 (0.60, 0.89) | 0.0017 |
| **Ventilatory Days** | TLV2 | 83 | 0.46 (0.39, 0.55) | <.0001 |
|  | O_E_LHR_2 | 84 | 0.47 (0.40, 0.56) | <.0001 |
|  | LHR2 | 84 | 0.48 (0.40, 0.57) | <.0001 |
|  | LHR1 | 76 | 0.49 (0.40, 0.61) | <.0001 |
|  | O_E_TLV_2 | 83 | 0.50 (0.41, 0.60) | <.0001 |
|  | O_E_LHR_1 | 76 | 0.50 (0.40, 0.61) | <.0001 |
|  | O_E_TLV_1 | 73 | 0.51 (0.41, 0.63) | <.0001 |
|  | PPLV1 | 73 | 0.52 (0.42, 0.64) | <.0001 |
|  | TLV1 | 73 | 0.61 (0.46, 0.80) | 0.0004 |
|  | PPLV2 | 83 | 0.63 (0.54, 0.73) | <.0001 |

**Table 4** Generalized logistics regression models or categorical outcomes

| Outcome | Measure | Comparison | n | Odds Ratio (95% CI) | p-value | Overall p-value |
| --- | --- | --- | --- | --- | --- | --- |
| **Chronic Lung Disease** | O_E_LHR_2 | Mild vs None | 75 | 0.49 (0.27, 0.91) | 0.0235 | 0.0008 |
|  | O_E_LHR_2 | Moderate/Severe vs None | 75 | 0.07 (0.02, 0.29) | 0.0003 | 0.0008 |
|  | LHR1 | Mild vs None | 65 | 0.45 (0.22, 0.92) | 0.0292 | 0.0023 |
|  | LHR1 | Moderate/Severe vs None | 65 | 0.10 (0.03, 0.38) | 0.0006 | 0.0023 |
|  | LHR2 | Mild vs None | 75 | 0.65 (0.37, 1.15) | 0.1417 | 0.0027 |
|  | LHR2 | Moderate/Severe vs None | 75 | 0.09 (0.02, 0.36) | 0.0006 | 0.0027 |
|  | TLV2 | Mild vs None | 73 | 0.67 (0.38, 1.16) | 0.1534 | 0.0039 |
|  | TLV2 | Moderate/Severe vs None | 73 | 0.11 (0.03, 0.40) | 0.0009 | 0.0039 |
|  | O_E_TLV_2 | Mild vs None | 73 | 0.58 (0.32, 1.03) | 0.0618 | 0.0078 |
|  | O_E_TLV_2 | Moderate/Severe vs None | 73 | 0.20 (0.07, 0.56) | 0.0021 | 0.0078 |
|  | O_E_LHR_1 | Mild vs None | 65 | 0.56 (0.27, 1.19) | 0.1334 | 0.0165 |
|  | O_E_LHR_1 | Moderate/Severe vs None | 65 | 0.22 (0.08, 0.62) | 0.0043 | 0.0165 |
|  | PPLV2 | Mild vs None | 73 | 0.87 (0.53, 1.43) | 0.5793 | 0.0312 |
|  | PPLV2 | Moderate/Severe vs None | 73 | 0.18 (0.05, 0.64) | 0.0086 | 0.0312 |
|  | TLV1 | Mild vs None | 62 | 0.61 (0.33, 1.13) | 0.1129 | 0.0453 |
|  | TLV1 | Moderate/Severe vs None | 62 | 0.26 (0.09, 0.77) | 0.015 | 0.0453 |
|  | O_E_TLV_1 | Mild vs None | 62 | 0.96 (0.48, 1.90) | 0.9001 | 0.1267 |
|  | O_E_TLV_1 | Moderate/Severe vs None | 62 | 0.38 (0.13, 1.08) | 0.0689 | 0.1267 |
|  | PPLV1 | Mild vs None | 62 | 1.21 (0.60, 2.42) | 0.5954 | 0.4097 |
|  | PPLV1 | Moderate/Severe vs None | 62 | 0.72 (0.28, 1.81) | 0.4824 | 0.4097 |
| **Pulmonary hypertension** | O_E_LHR_2 | Mild vs None | 75 | 0.24 (0.11, 0.55) | 0.0007 | 0.0003 |
|  | O_E_LHR_2 | Moderate/Severe vs None | 75 | 0.07 (0.01, 0.36) | 0.0013 | 0.0003 |
|  | LHR2 | Mild vs None | 75 | 0.29 (0.13, 0.63) | 0.0018 | 0.0008 |
|  | LHR2 | Moderate/Severe vs None | 75 | 0.10 (0.02, 0.43) | 0.0024 | 0.0008 |
|  | PPLV2 | Mild vs None | 73 | 0.23 (0.08, 0.64) | 0.005 | 0.0017 |
|  | PPLV2 | Moderate/Severe vs None | 73 | 0.06 (0.01, 0.38) | 0.0026 | 0.0017 |
|  | TLV2 | Mild vs None | 73 | 0.36 (0.17, 0.77) | 0.0089 | 0.0021 |
|  | TLV2 | Moderate/Severe vs None | 73 | 0.09 (0.02, 0.44) | 0.0026 | 0.0021 |
|  | O_E_LHR_1 | Mild vs None | 65 | 0.56 (0.30, 1.05) | 0.0713 | 0.0142 |
|  | O_E_LHR_1 | Moderate/Severe vs None | 65 | 0.19 (0.06, 0.62) | 0.0056 | 0.0142 |
|  | O_E_TLV_2 | Mild vs None | 73 | 0.54 (0.29, 1.02) | 0.056 | 0.0186 |
|  | O_E_TLV_2 | Moderate/Severe vs None | 73 | 0.24 (0.08, 0.72) | 0.0108 | 0.0186 |
|  | O_E_TLV_1 | Mild vs None | 62 | 0.57 (0.29, 1.11) | 0.0976 | 0.0186 |
|  | O_E_TLV_1 | Moderate/Severe vs None | 62 | 0.16 (0.04, 0.61) | 0.0073 | 0.0186 |
|  | PPLV1 | Mild vs None | 62 | 0.51 (0.27, 0.96) | 0.0385 | 0.037 |
|  | PPLV1 | Moderate/Severe vs None | 62 | 0.31 (0.10, 0.94) | 0.0384 | 0.037 |
|  | LHR1 | Mild vs None | 65 | 0.70 (0.40, 1.24) | 0.2268 | 0.0391 |
|  | LHR1 | Moderate/Severe vs None | 65 | 0.20 (0.06, 0.72) | 0.0133 | 0.0391 |
|  | TLV1 | Mild vs None | 62 | 0.71 (0.41, 1.24) | 0.2256 | 0.091 |
|  | TLV1 | Moderate/Severe vs None | 62 | 0.26 (0.07, 0.95) | 0.0408 | 0.091 |
